# Reproducibility of Post-Amphetamine [^11^C]FLB 457 Binding to Cortical D_2/3_ Receptors

**DOI:** 10.1371/journal.pone.0076905

**Published:** 2013-09-30

**Authors:** Rajesh Narendran, Michael Himes, N. Scott Mason

**Affiliations:** Department of Radiology, University of Pittsburgh, Pittsburgh, Pennsylvania, United States of America; Chiba University Center for Forensic Mental Health, Japan

## Abstract

In a recent positron emission tomography (PET) study, we demonstrated the ability to measure amphetamine-induced dopamine (DA) release in the human cortex with the relatively high affinity dopamine D_2/3_ radioligand [^11^C]FLB 457. Herein we report on reproducibility and reliability of [^11^C]FLB 457 binding potential relative to non-displaceable uptake (BP_ND_) following an acute amphetamine challenge. Ten healthy human subjects were studied twice with [^11^C]FLB 457 following an acute amphetamine (oral, 0.5 mg kg^-1^ dose) challenge on two-separate days approximately one week apart. D_2/3_ receptor binding parameters were estimated using a two-tissue compartment kinetic analysis in the cortical regions of interest and cerebellum (reference region). The test-retest variability and intraclass correlation coefficient were assessed for distribution volume (V_T_), binding potential relative to plasma concentration (BP_P_), and BP_ND_ of [^11^C]FLB 457. The test-retest variability of [^11^C]FLB 457 V_T_, BP_P_ and BP_ND_ were ≤ 17%, 22% and 11% respectively. These results, which are consistent with the published test-retest variability for this ligand measured under baseline conditions demonstrate that the post-amphetamine [^11^C]FLB 457 BP_ND_ is reproducible. These data further support the use [^11^C]FLB 457 and amphetamine to characterize cortical dopamine transmission in neuropsychiatric disorders.

## Introduction

The competition between dopamine (DA) and D_2/3_ radiotracer binding (such as [^11^C]raclopride and [^123^I]IBZM) following an amphetamine challenge is a noninvasive measure of the change in extracellular DA induced by the challenge [1-7] and is thought to provide information as to the status of DA transmission in the brain. Previous investigations have used this technique to report abnormal DA release in the striatum in patients with schizophrenia [4,8], alcoholism [9] and drug abuse [10-12]. A limitation of these previous studies is that the relatively low binding potential for [^11^C]raclopride and [^123^I]IBZM in the extrastriatal regions precluded the investigation of DA in the cortical regions that have been implicated in these disorders. Thus, it is of interest to develop an imaging paradigm to measure cortical DA in schizophrenia and addiction. In a recent PET study, we demonstrated the ability to detect amphetamine-induced DA release in the human cortex with the dopamine D_2/3_ radioligand [^11^C]FLB 457 [13]. The results of this study, which showed a significant reduction in the *in vivo* binding of [^11^C]FLB 457 following oral amphetamine (0.5 mg kg^-1^) led to further characterization of this imaging paradigm as a tool to measure cortical DA release. In a series of validation studies following this report we have shown: good reproducibility for [^11^C]FLB 457 BP_ND_ under baseline conditions [14]; no carryover mass induced decrease in BP_ND_ [14]; a relatively small fraction of D_2/3_ receptor specific binding for [^11^C]FLB 457 in the cerebellar reference region [15]; and a linear relationship between the amphetamine-induced decrease in [^11^C]FLB 457 BP_ND_ and increase in extracellular DA using combined PET and microdialysis [16]. In addition, we have replicated our initial report of amphetamine-induced displacement of [^11^C]FLB 457 BP_ND_ in an independent cohort of subjects [17]. In a previous study, we demonstrated that the test-retest variability for [^11^C]FLB 457 BP_ND_ measured under control conditions (i.e., at baseline) is an acceptable ≤ 15% in the cortical regions of interest -- a result which is also consistent with other published [^11^C]FLB 457 reproducibility studies [14,18,19]. Here, we were interested in evaluating the test-retest variability of the post-amphetamine [^11^C]FLB 457 BP_ND_ to ensure that it is reproducible. To evaluate this issue we conducted test and retest amphetamine studies to measure the reproducibility of the post-amphetamine V_T_ (regional distribution volume), BP_P_ and BP_ND_ in ten healthy human subjects.

## Materials and Methods

### Ethics statement

The Institutional Review Board and Radioactive Drug Research Committee of the University of Pittsburgh approved the study. All subjects provided written informed consent.

### Study design

A total of 20 PET scans were acquired for this study in ten healthy control (4 females/6 males; 2 Asian/8 Caucasian; age 23 ± 4, weight 70 ± 12 kg) subjects. Each subject was scanned with [^11^C]FLB 457 following amphetamine (oral, 0.5 mg kg^-1^) in a test and retest condition separated by one week.

### PET Protocol

Radiolabeling of [^11^C]FLB 457 was performed as outlined in previously published procedures [20]. Imaging experiments with amphetamine were conducted on the Siemens ECAT EXACT HR+ scanner consistent with previously described image acquisition protocols [13]. [^11^C]FLB 457 was administered as a bolus intravenous injection three-hours following the administration of 0.5 mg kg^-1^ of d-amphetamine (Dexedrine, oral formulation) and emission data were collected for 90 minutes. An oral amphetamine dose of 0.5 mg kg^-1^ is consistent with what has been used in previous PET investigations to measured dopamine release in the striatal and extra striatal regions [21-25]. Previous microdialysis studies in primates have shown that 0.5 mg kg^-1^ of amphetamine increases extracellular dopamine concentrations by 1320 ± 432% [16].

Following radiotracer injection, arterial samples were collected manually approximately every 6 seconds for the first 2 minutes and thereafter at longer intervals. A total of 35 samples were obtained per scan. Following centrifugation, plasma was collected in 200 µL aliquots and activities were counted in a gamma well counter. To determine the plasma activity representing unmetabolized [11C]FLB 457 parent compound, six samples (collected at 4, 10, 20, 40, 60 and 80 min) were further processed using high-performance liquid chromatography methods [26] and fitted using a Hill model [27,28]. The input function was then calculated as the product of total counts and interpolated parent fraction at each time point. The measured input function values were fitted to a sum of three exponentials from the time of peak plasma activity and the fitted values were used as the input to the kinetic analysis. The clearance of the parent compound (C_L_, L/h) was calculated as the ratio of the injected dose to the area under the curve of the input function [29]. In addition, measurement of plasma free fraction (f_P_) for [^11^C]FLB 457 was performed [30]. Amphetamine plasma levels were measured in three arterial samples obtained at time 0 min, 45 min and 90 relative to the PET scan as previously described [31]. These data ensured that differences in plasma amphetamine concentration did not bias the test and retest comparison.

### MRI Protocol

Prior to PET imaging, a magnetization prepared rapid gradient echo structural MRI scan was obtained using a Siemens 3 Tesla Trio scanner for determination of regions of interest. MRI segmentation was performed using the automated segmentation tool [32] implemented in the FMRIB Software Library v4.0 [33].

### Analysis of PET data

PET data were reconstructed and processed with the image analysis software MEDx (Sensor Systems, Inc., Sterling, Virginia) and SPM2 (www.fil.ion.ucl.ac.uk/spm) as described in [13]. Frame-to-frame motion correction for head movement and MR-PET image alignment were performed using a mutual information algorithm implemented in SPM2. Time activity curves were generated for the eight cortical regions of interest and cerebellum (reference region) using the criteria and methods outlined in [13,14]. Sampled cortical regions (n = 8) included the medial temporal lobe (MTL), anterior cingulate cortex (ACC), dorsolateral prefrontal cortex (DLPFC), orbital frontal cortex (OFC, defined using criteria outlined in Lacerda 2003), medial prefrontal cortex (MPFC), temporal cortex (TC), parietal cortex (PC), and occipital cortex (OC). The three outcome measures provided are regional tissue distribution volume (VT, mL cm-3), binding potential relative to plasma concentration (BPP, mL cm-3) and binding potential relative to non-displaceable uptake (BPND, unitless) [34]. Derivation of [11C]FLB 457 VT in the regions of interest and cerebellum were performed using a two-tissue compartment kinetic analysis using the arterial input function as described in [13].

### Statistical analysis

The reproducibility of the plasma (f_p_, C_L_ and amphetamine levels) and brain (V_T_, BP_P_ and BP_ND_) outcome measures were evaluated for their *variability* and *reliability*.

The test-retest variability (VAR) was calculated as the absolute value of the difference between the test and retest, divided by the mean of the test and retest values.

To evaluate the within-subject variability relative to the between-subject variability, both within-subject standard deviation (WSSD) and between-subject standard deviation (BSSD) were calculated and expressed as fraction of mean value (WS CV and BS CV). The reliability of the measurements was assessed by the intraclass correlation coefficient (ICC) calculated as [35]:

$\frac{BSMSS–WSMSS}{BSMSS+\left(n–1\right)WSMSS}$

where BSMSS is the mean sum of square between subjects, WSMSS is the mean sum of square within subjects and n is the number of repeated observations (n = 2 in this study). This statistic estimates the relative contributions of between and within subject variability and assumes values from -1 (i.e. BSMSS = 0) to 1 (identity between test and retest, i.e. WSMSS = 0).

## Results

### Baseline scan parameters

The mean injected dose for [^11^C]FLB 457 in the test and retest conditions were 8.4 ± 0.3 mCi and 7.5 ± 1.4 mCi. The mean injected specific activity in the test and retest conditions were 10186 ± 3638 Ci/mmol and 7805 ± 3762 Ci/mmol. The mean injected mass for [^11^C]FLB 457 in the test and retest conditions were 0.3 ± 0.1 µg and 0.4 ± 0.1 µg. There were no significant differences in injected dose or mass between the test and the retest conditions (paired t test, p > 0.05).

### Plasma analysis

The mean [^11^C]FLB 457 fp, C_L_, and amphetamine plasma levels (measured at 0, 45 and 90 min following the [^11^C]FLB 457 injection) and their corresponding VAR and ICC are provided in Table 1.

**Table 1:** Reproducibility of [^11^C]FLB 457 and amphetamine in plasma.

Parameter	Mean	BSSD	BSSD CV	WSSD	WSSD CV	VAR + SD	ICC
[^11^C]FLB 457 fp (%)	0.38	0.05	0.14	0.06	0.15	23.0% ± 18.6%	-0.05
[^11^C]FLB 457 Clearance (L/h)	69.72	14.33	0.21	8.85	0.13	23.7% ± 15.3%	0.45
Amphetamine level 0 min (ng/mL)	87.34	11.83	0.14	4.11	0.05	8.1% ± 7.2%	0.78
Amphetamine level 45 min (ng/mL)	76.60	11.22	0.15	3.45	0.05	7.1% ± 6.6%	0.83
Amphetamine level 90 min (ng/mL)	73.82	11.08	0.15	4.16	0.06	9.8% ± 8.8%	0.75

Values are the mean of 10 subjects with each value measured twice. BSSD CV = between subject standard deviation coefficient of variation, WSSD CV = within subject standard deviation coefficient of variation, VAR = test/retest variability, ICC = intraclass correlation coefficient.

### Brain analysis

The mean VT, BP_P_, BP_ND_ and their corresponding VAR and ICC for the regions of interest are provided in Tables 2, 3 and 4.

**Table 2:** Reproducibility of post-amphetamine [^11^C]FLB 457 total distribution volume (V_T_, mL cm^-3^).

Region	Mean	BSSD	BSSD CV	WSSD	WSSD CV	VAR + SD	ICC
Cerebellum	3.90	0.51	0.13	0.37	0.09	14.7% ± 11.1%	0.32
Medial Temporal Lobe	8.45	1.55	0.18	0.79	0.09	14.9% ± 11.1%	0.59
Anterior Cingulate Cortex	6.97	1.15	0.16	0.68	0.10	14.7% ± 13.0%	0.48
Dorsolateral prefrontal Cortex	5.96	1.32	0.22	0.54	0.09	14.4% ± 10.7%	0.72
Orbital Frontal Cortex	7.09	1.49	0.21	0.72	0.10	16.8% ± 11.7%	0.62
Medial Prefrontal Cortex	6.36	1.06	0.17	0.54	0.09	13.5% ± 11.1%	0.59
Temporal Cortex	9.64	2.30	0.24	0.90	0.09	14.8% ± 11.3%	0.73
Parietal Cortex	6.13	1.61	0.26	0.54	0.09	14.0% ± 10.8%	0.80
Occipital Cortex	5.81	1.59	0.27	0.53	0.09	14.7% ± 10.7%	0.80

Values are the mean of 10 subjects with each value measured twice. BSSD CV = between subject standard deviation coefficient of variation, WSSD CV = within subject standard deviation coefficient of variation, VAR = test/retest variability, ICC = intraclass correlation coefficient.

**Table 3:** Reproducibility of post-amphetamine [^11^C]FLB 457 binding potential relative to plasma concentrations (BP_P_, mL cm^-3^).

Region	Mean	BSSD	BSSD CV	WSSD	WSSD CV	VAR + SD	ICC
Medial Temporal Lobe	4.55	1.20	0.26	0.45	0.10	16.1% ± 11.1%	0.75
Anterior Cingulate Cortex	3.07	0.74	0.24	0.32	0.11	16.9% ± 14.5%	0.68
Dorsolateral prefrontal Cortex	2.06	0.91	0.44	0.19	0.09	14.8% ± 9.3%	0.92
Orbital Frontal Cortex	3.19	1.08	0.34	0.38	0.12	21.7% ± 13.4%	0.78
Medial Prefrontal Cortex	2.46	0.69	0.28	0.18	0.08	12.4% ± 11.1%	0.87
Temporal Cortex	5.74	1.90	0.33	0.56	0.10	15.3% ± 11.3%	0.84
Parietal Cortex	2.23	1.21	0.54	0.18	0.08	13.9% ± 9.8%	0.96
Occipital Cortex	1.91	1.17	0.61	0.19	0.10	17.2% ± 9.8%	0.95

Values are the mean of 10 subjects with each value measured twice. BSSD CV = between subject standard deviation coefficient of variation, WSSD CV = within subject standard deviation coefficient of variation, VAR = test/retest variability, ICC = intraclass correlation coefficient.

**Table 4:** Reproducibility of post-amphetamine [^11^C]FLB 457 binding potential relative to non specific uptake (BP_ND_, unitless).

Region	Mean	BSSD	BSSD CV	WSSD	WSSD CV	VAR + SD	ICC
Medial Temporal Lobe	1.16	0.26	0.22	0.06	0.05	6.8% ± 5.8%	0.91
Anterior Cingulate Cortex	0.78	0.15	0.19	0.03	0.04	6.9% ± 5.2%	0.91
Dorsolateral prefrontal Cortex	0.52	0.19	0.37	0.02	0.03	5.4% ± 4.9%	0.98
Orbital Frontal Cortex	0.81	0.21	0.27	0.05	0.06	11.1% ± 7.9%	0.90
Medial Prefrontal Cortex	0.63	0.15	0.24	0.02	0.03	4.3% ± 4.3%	0.97
Temporal Cortex	1.45	0.36	0.25	0.05	0.03	3.9% ± 3.9%	0.97
Parietal Cortex	0.55	0.26	0.47	0.01	0.03	4.2% ± 2.8%	0.99
Occipital Cortex	0.47	0.26	0.56	0.02	0.05	6.7% ± 4.9%	0.99

Values are the mean of 10 subjects with each value measured twice. BSSD CV = between subject standard deviation coefficient of variation, WSSD CV = within subject standard deviation coefficient of variation, VAR = test/retest variability, ICC = intraclass correlation coefficient.

## Discussion

The results of this study show that the post-amphetamine [^11^C]FLB 457 BP_ND_ is reproducible. The test-retest variability of ≤ 15% for [^11^C]FLB 457 BP_ND_ in the cortical regions of interest measured in the post-amphetamine condition is comparable to that reported in the baseline condition in our previous report [14]. It was necessary to evaluate the test-retest variability for the post-amphetamine PET measurements because [^11^C]FLB 457 BP_ND_ is lower following amphetamine compared to baseline [13]. The good reproducibility of [^11^C]FLB 457 BP_ND_ in the baseline and post-amphetamine conditions suggest that the relatively low cortical binding potential in itself does not pose a problem to the use of this tool to measure cortical DA release. This point is further illustrated in Table 5 which shows an effect size (d) of 0.5 to 2.2 to measure amphetamine-induced change (Δ) of [^11^C]FLB 457 BP_ND_ in the cortical regions. These d values are comparable to that observed with [^11^C]raclopride to detect of an effect for amphetamine in the striatal subdivisions (d=0.8 to 1.9, derived as ΔBPND/test-retest variability using data in [36,37]). This suggests that the [^11^C]FLB 457 BP_ND_ measured under baseline and post-amphetamine conditions will be distinguishable.

**Table 5:** Effect size to measure amphetamine-induced displacement of [^11^C]FLB 457 BP_ND_.

Region	Δ BP_ND_ (%)	BASE T-RT (%)	Post-AMPH T-RT (%)	Effect size (d)
Medial Temporal Lobe	-7 ± 6	11 ± 5	7 ± 6	0.76
Anterior Cingulate Cortex	-8 ± 8	15 ± 8	7 ± 5	0.68
Dorsolateral prefrontal Cortex	-13 ± 15	8 ± 6	5 ± 5	1.95
Orbital Frontal Cortex	-8 ± 15	7 ± 6	11 ± 8	0.87
Medial Prefrontal Cortex	-11 ± 14	6 ± 4	4 ± 4	2.16
Temporal Cortex	-4 ± 9	10 ± 6	4 ± 4	0.53
Parietal Cortex	-12 ± 13	8 ± 4	4 ± 3	1.90
Occipital Cortex	-5 ± 20	10 ± 4	7 ± 5	0.58

% values shown are mean ± standard deviation (SD); Δ BP_ND_ is amphetamine-induced displacement of [^11^C]FLB 457 BP_ND_ [13,15];

BASE T-RT is test-retest variability [^11^C]FLB 457 BP_ND_ under baseline conditions [14];

Post-AMPH T-RT is test-retest variability of [^11^C]FLB 457 BPND under post-amphetamine conditions (this study, Table 4);

Effect size (d) is computed as mean Δ BP _ND_/ mean pooled variability; Pooled variability was calculated as the square root of (BASE T-RT^2^ + POST-AMPH T-RT^2^)/2 to incorporate both the baseline and post-amphetamine test-retest data.

The measured test-retest variability for V_T_ (14-17%) and BP_P_ (12-22%) was higher than BP_ND_ (4-11%) in the post-amphetamine condition. This is consistent with what has been reported for [^11^C]FLB 457 in the baseline condition, and other DA D_2/3_ PET radioligands such as [^11^C]raclopride and [^11^C]NPA [38-40]. BP_ND_ as opposed to BP_P_ and V_T_ is associated with lower test-retest variability because it is less vulnerable to the experimental errors associated with the measurement of the plasma input function. Therefore, it is the preferred outcome measure in amphetamine challenge studies that measure a relatively small decrease in radiotracer binding (^~^10-15%) [41]. An important assumption in the use of ΔBP_ND_ to quantify dopamine release is that amphetamine does not affect the non-specific binding in the brain (V_ND_). This assumption is tested in amphetamine-PET studies by documenting V_ND_ in the baseline and post-amphetamine condition. The use of ΔBP_P_ and ΔV_T_ to quantify dopamine release is necessary when this assumption fails because these outcome measures are somewhat less influenced by amphetamine-induced changes in V_ND_ [42]. Thus, it was necessary to document the test-retest variability for all outcome measures -- BP_ND_, BP_P_ and V_T_ in the post-amphetamine condition. These results suggest that the use of ΔBP_P_ and ΔV_T_ to quantify dopamine release in amphetamine challenge studies might be limited by its relatively higher test-retest variability.

In summary, we evaluated the reproducibility of the post-amphetamine [^11^C]FLB 457 BP_ND,_ and found it to be consistent with that measured under baseline conditions. The results of this reproducibility study support the use of [^11^C]FLB 457 to measure cortical dopamine release despite its relatively low binding potential (BP_ND_).
